# The Fourth Annual Report of the Registrar-General of Births, Deaths, and Marriages in England, 1842

**Published:** 1844-07

**Authors:** 


### Art. XVI.

1. *The Fourth Annual Report of the Registrar-general of Births, Deaths, and Marriages in England*, 1842.
2. *The Fifth Annual Report of the Registrar-general of Births, Deaths, and Marriages in England*, 1843.
3. *An Essay on the best modes of representing accurately, by Statistical Returns, the Duration of Life, and the Pressure and Progress of the Causes of Mortality amongst different classes, &c.* By Edwin Chadwick, Esq. 8vo, pp. 42. (*Reprinted from the Quarterly Journal of the Statistical Society, April,* 1844.)

The Fourth Annual Report of the Registrar-general contains the usual tables, showing the movement of the population, both absolutely, during the year ending June 30th, 1841, and comparatively, with reference to the preceding year. It appears, that the number of deaths was greater in 1840-1 than in the previous year; the mortality only slightly exceeded that of the antecedent year, but was nearly 6 per cent. (5·71) above that of the year 1838-9.

In the Appendix, by Mr. Farr, we have several interesting documents, displaying his usual industry and research. The first is an essay on the increase of population, fully corroborating those anti-Malthusian views of Mr. Alison, to which we have given (in promulgating them,) our cordial support. In about fifty years, the population of Great Britain will have doubled, and must be supplied with subsistence; but (to quote Mr. Farr's words) there will also be double the number of men to create subsistence for her families, to man her fleets, to defend her inviolate hearths, to work her mines and manufactories, to extend her commerce, to open new regions of colonization ; and double the number of minds to discover new truths, to confer the benefits, and to enjoy the felicity of which human nature is susceptible. In this little essay, Mr. Farr, like a warm-hearted patriot, draws quite an arcadian picture of his country. There can be no harm done by inspiring cheerfulness and hope, but we see dark shadows, and sad stains on the picture, such as our author depicts not.

The next document is on the registration of the causes of death, and contains the statistical nosology placed in the hands of the district registrars for their guidance; to it is appended a useful alphabetical list of the diseases and their synonyms. Then follows an essay on nomenclature, including an analysis of morbid phenomena, highly creditable, we think, to Mr. Farr, for we know quite well, easy as they read, how difficult arrangements of this kind are. Numerous new terms are also recommended for adoption, and some of questionable value. It is proposed to state of a person poisoned by arsenic, that he died of *arsenicia*, or by tobacco, of *nicotinia.* People dying from cold are to be registered as dying of *psychria*, from of starvation, *pinia.* In like manner, Mr. Farr has accommodated the diseases arising from a specific animal poison, with new and not inconvenient names, excepting that they are new. The contagious matter of syphilis is to be called syphiline ; of cowpox, vaccinine ; glanders, equinine ; measles, rubeoline, &c. "The existence of gangrenine, ergotine, tetanine, &c. may also be admitted." We need scarcely say that none of these names have much chance of being speedily "admitted" into medical glossology. One new term will, however, survive. A single word, as Mr. Farr observes, is required to replace the long periphrasis, "epidemic, endemic, and contagious diseases." This want he has well supplied by the terms *zymosis* and *zymotic*, from the Greek word ζυμόω, to ferment. The term is strikingly applicable, independently of the zymotic theory, to those diseases, in which a small portion of a contagious poison introduced into the system multiplies itself, and leavens the whole lump. We gladly call all epidemic, endemic, and contagious diseases, zymotic, and shall at once adopt the term.

The Fifth Annual Report is the commencement of a new series of abstracts, extending over the ordinary year ending December 31st, instead of a year ending 30th of June. This is therefore the report for 1841. The census having been taken in that year, and thus extensive data afforded for statistical estimates and comparisons, the Registrar-general has availed himself of that circumstance, and rendered this report more valuable than the preceding, by numerous additional tables. One of the most important of these additions is the essay on life-tables generally, containing a life-table for England, deduced from official returns. Life-tables, and diagrams illustrative of these, are given to exhibit the relative healthiness of three districts in London, and in Liverpool. A good deal of controversial discussion is introduced, and somebody written at very vigorously without mentioning names; but we suppose that Mr. Chadwick is the person referred to, and that the "fallacies" in the Appendix to his Supplementary Report on Interment, are denounced by the Registrar-general. We observe that in the essay by Mr. Chadwick, additional proofs are given of his doctrine, that the number of inhabitants living to one death does not express the average duration of life so accurately as the average age at death. Without entering into the merits of the controversy, we can easily imagine that some degree of irritation may be excited by this intrusion of Mr. Chadwick into the domain of the actuaries, whom it is quite possible that Mr. Chadwick may set right in some particulars.

Since writing the above, we have seen a paper by Mr. Neison, an actuary in an insurance office, controverting Mr. Chadwick's views. It is laid down by the author, firstly, that the average age at death is regulated by the distribution of the population as to age; and secondly, that this distribution is not uniform over the various periods of life in any two places, not even approximatively. The first proposition is a self-evident truth; the average age of the dying must necessarily have a fixed ratio to that of the living; if a large per cent. be young, the average age will be low, and vice versâ? No elaborate tables were required to prove this. With regard to the second proposition, we have to observe that Mr. Neison's tables, nay, his actual assertions, flatly contradict it. "While," he says, "in the counties of Essex, Suffolk, Norfolk, and Hereford, there is on an average 8·864 per cent. of the population above sixty years of age, in the towns of Liverpool, Sheffield, Manchester, Birmingham, Leeds, and Exeter, there is only 4·6 per cent. alive above the same age. These irregularities will be found to prevail in any other period of life that may be selected." It is manifest that what he calls irregularities, are, in fact, *uniform* results of the difference of health between town and rural life, as the following shows. Of persons aged from forty to sixty years, there were living in

| | per cent. | | per cent. |
|---|---|---|---|
| Exeter | 13·28 | Liverpool | 14·87 |
| Sheffield | 15·50 | Manchester | 15·43 |
| Birmingham | 15·15 | Leeds | 15·23 |

The numbers for the four manufacturing towns, Sheffield, Birmingham, Manchester, and Leeds, are remarkably uniform, indeed nearly the same; the slight differences in Exeter and Liverpool depend, no doubt, on local circumstances.

The four agricultural counties exhibit a similar uniformity. Of persons aged from forty to sixty, there are in

| | per cent. | | per cent. |
|---|---|---|---|
| Devon | 16·97 | Essex | 16·27 |
| Norfolk | 16·50 | Suffolk | 15·98 |

Can anything display the different effects of rural and town life, on longevity, more uniformly, more regularly, or more strikingly? Mr. Neison must have a singularly dull perception not to see that his own facts are as strongly corroborative of the views he attempts to controvert, as facts of the kind can possibly be. We might criticise other of Mr. Neison's deductions, as justly as the preceding, if they were really worth the criticism; but he has manifestly quite misunderstood the scope of Mr. Chadwick's arguments, and the nature of his facts.

It appears from the calculations in this, the Fifth Report, that the probable duration of life in England is 45½ years; the probable life of a male infant is 44, of a female 47 years. The expectation of life in England generally is 41 years; for males, 40 years, females, 42 years. We regret that we cannot find room for a most interesting table, exhibiting the expectation of life at different ages, and in the two sexes. Mr. Farr's Appendix in this Report surpasses his preceding contributions, and will add to his already high reputation.

We shall not follow our previous plan, and make copious extracts from the Report, as we trust the original is by this time in the hands of our readers; but devote the space thus saved to setting forth some of the advantages to society, and increments to science that these national records may afford, comparing them with those that *are* afforded.

One of the proposed advantages of the new system was the greater accuracy of registering the fact of birth, marriage, or death, and the more perfect prevention of fraud. It does not appear, however, that the act has been so successfully applied in this respect as it might be; nor, under the existing regulations, is it capable of that successful application. In registering a death, for instance, the person who testifies to have been present at the death, may *possibly* be the identical individual whose supposed death is registered: but we admit that this is not very likely to occur. The bill contains, in fact, no machinery whatever for identifying a corpse.

It must be admitted, also, that the new arrangements are not calculated to ascertain *the cause of death*, in a certain manner, but only approximatively. This is generally admitted by medical men; and they are naturally led to form this opinion by seeing so many difficulties in the way of ascertaining the real cause of death. From personal experience they know quite well that, in numerous instances, it is really impossible to state of what precise disease the individual died, or to name the disease at all. Then, again, there is often such a complication of disease in the same individual that four or five might be distinctly mentioned. Thus, a person may have chronic diarrhea, albuminuria, hypertrophy of the heart, anasarca, bronchitis, and paraplexy. Again, practitioners often treat symptoms, and will name a disease from a leading symptom, so that in the case of the individual just supposed, the cause of death might after all be entered as asthma, or orthopnea, or hydrothorax, according to the whim or theory of the medical attendant. But, in very many cases, no medical opinion whatever is given as to the cause of death The circular letter of the colleges was literally a dead letter; we should almost suspect, indeed, that it did more harm than good, as practitioners generally felt, that by acting on the instructions issued, they would acknowledge the authority of a body whose right was contested, and with which they had no interests in common. Consequently, in a certain proportion of cases, the nurse or friends, or the messenger sent to the registrar states the cause of death as it appears to them, and with what accuracy may be readily surmised. While, therefore, we admit that the present tables are infinitely superior to those that could be constructed before the passing of the act; and, also, that they are valid for many of the purposes to which they are applied by the Registrar-general, we can only regard them, in a great number of cases, as approximations, not positive results. Under the present system it is manifest that much time, labour, and zeal are being wasted, and that before this important measure can be put into really efficient operation, Mr. Chadwick's suggestion must be acted on, namely, to have an inspection of every corpse by a competent, and, of course, medical officer.

Supposing, however, this method be adopted, and more accurate returns secured, are the tabular arrangements of the Registrar-general such as to secure to medical science all the advantages to be extracted from these returns? Or do they even attain the objects for which they are drawn up? These objects are truly stated, and there is no want of liberal views, as will be seen by the subjoined extract:

"Every person is naturally most interested in the district in which he resides; and the present form of publication will afford the scientific inquirer the means of framing tables of mortality; of calculating the mean duration of life in his own district, and comparing the results with those deduced from the facts registered in neighbouring districts, or in the whole kingdom. The inhabitants will thus learn, from authentic documents, the relative sanitary state of their neighbourhood; the causes of disease will be discovered, and suggestions which may lead to numerous improvements will be pressed upon the attention of the resident proprietors and authorities. The friendly societies will obtain from the local tables the means of adjusting their premiums equitably to the prevailing rate of sickness and mortality, which may be a fourth or a half higher in some districts than in others, and will be found to differ among the same classes, so as to affect materially the money-value of assurance, and the stability and prosperity of the societies. Materials of thought and guides of action will thus be afforded, on questions affecting health, life, and death, to scientific and benevolent persons in all parts of the kingdom." (Fourth Annual Report, p. 3.)

These remarks refer to forty-six pages of closely-printed abstracts of ages of deaths, in 324 districts, and to tables of the proportions of births, marriages, and deaths in England and Wales, arranged in counties. Now, what do these tables really teach us? Take a district with an agricultural and a town population, and they teach us, probably, that the mortality is equal to the mean mortality of England and Wales, a fact well enough known already; or take a civic population, as that of Liverpool, for example, and they show very clearly, (or at least, when illustrated by the Registrar-general's diagrams,) that the sanitary condition of Liverpool is lower than that of Surrey, or of the metropolis; but this fact we had learnt already by the simpler process of dividing the population by the annual deaths; the diagrams are mute as to the detailed information wanted. Will the Registrar-general say that the life-insurance societies are to frame their life-tables for Liverpool on these data, and demand a higher premium on the healthy lives in that wealthy emporium, than in London or Surrey? or that the benefit societies are to alter their rates in Liverpool? Manifestly not, because we know that the increased mortality is not among the life-insuring and provident classes, but among the immigrant Irish, (one third of the population of Liverpool is Irish,) and among the poor people living in the damp, unsewered cellars and courts of the town.

To place the Registrar-general's efforts in the proper light, they must be considered to be, what they are manifestly intended to be, namely, contributions to medical topography ; and, valuable as they are, so far as they go, it is manifest, that in this point of view they are extremely defective. This is no fault of the Registrar-general's ; but it *is* one, and one of such magnitude, as must render abortive his best efforts, until it is amended. In questions of this kind, it is obvious that meteorological changes, the nature of the climate and the soil, the composition of the air and the water, the food, habitations, and employments of the people, their social habits, morals, and even their race, (as in the example of Liverpool,) are all to be taken into account. Viewed in this light, the annual reports (with certain exceptions) supply no precise or available data. Let us take meteorological changes, the first of the subjects mentioned. During one complete year, there are a variety of meteorological phenomena ; at one time, hurricanes, at another meteors, at another, a succession of violent thunder-storms, at another, a comet, or shocks of earthquakes, or unnatural changes of temperature ; if we inquire what influence these had on the health or mortality of the people, and look to the Annual Reports for an answer, we may look in vain. There are not even the rough materials of an inquiry, excepting in the metropolitan weekly returns, and these are scarcely sufficient for the purpose in their present state. Nor can we learn accurately so simple a point as the influence of temperature or of the *seasons*, properly so called, on health. It is true, the Registrar-general professes to give this knowledge. Thermometric and barometric observations are detailed, and a long series of most elaborate tables drawn up, showing the births, marriages, and deaths in the four quarters of the year. Conclusions are deduced from these data as follows :

"The greatest number of marriages (36,542) occur in autumn, and the smallest number (25,174) in winter : the difference between these extremes is 11,368 ; and the four seasons stand in the following order :

| | *Winter.* | *Summer.* | *Spring.* | *Autumn.* |
|---|---|---|---|---|
| | January, February, March. | July, August, September. | April, May, June. | October, November, December. |
| Average, 1839-41 | 25,174 | 29,502 | 31,559 | 36,542 |

. . . . The marriages in the four quarters are therefore the terms of a proportion." (Fifth Annual Report, p. v.)

Similar calculations and results are stated with respect to the births. We extract the following tables of seasonal mortality.

"If the mortality were uniformly at the same rate as in winter, 391,059 deaths would happen annually ; if at the same rate as in summer, 302,827 deaths would be registered. This exhibits in a striking light the fatal effects of cold ; and also of the crowding and privations to which a considerable part of the population is more exposed in cold than in warm weather. The average corrected number of deaths in the seasons would be :

| *Winter.* | *Spring.* | *Summer.* | *Autumn.* |
|---|---|---|---|
| 97,765 | 89,141 | 75,707 | 83,639 |

" By transposing the autumn and summer terms, the law is discovered which has regulated the mortality of the seasons. Thus :

| | *Winter.* | | *Spring.* | | *Autumn.* | | *Summer.* |
|---|---|---|---|---|---|---|---|
| | 97,765 | : | 89,141 | :: | 83,639 | : | 75,707 |
| Differences | | 7629 | | 4146 | | 8333 | |

"The terms are in proportion: the product of the two extremes is nearly equal to the product of the middle terms; or the deaths in winter are to the deaths in spring as the deaths in autumn are to those in summer." (Fifth Annual Report, p. x.)

Now, on these statements we would remark, that they may be, and no doubt are, perfectly accurate as regards the four *quarters* of the year; but they cannot be received as even approximations to the relative health and mortality of the *seasons.* We would ask the Registrar-general how March can be called a winter month, when everybody knows that it is the opening month of spring? Or how can September be summer, when it is the veritable type of autumn, with its ripe fruit and corn? Or how is December an autumnal month, when all the world knows that Christmas-day is in mid-winter—the poetical personification of Yale being, indeed, an old man with a beard of long icicles? What, then, is the inference? So far as giving information on the pathological or physiological influence of the *seasons*, properly so called, all these laboriously-educed results are useless; the law of proportion a nullity; and the elaborate tables of births, marriages, and deaths in the four "quarters," and the three hundred and twenty-four districts, of no value.

We have had the deaths in the metropolis from four causes, selected from the weekly tables of mortality, and arranged according to the Registrar-general's plan, and according to the natural division of the year, as marked by the solstices and equinoxes. The months which make up the season of the Registrar-general may be seen *ante:* according to our arrangement, winter comprises the three last weeks in November, the whole of December and January, and the first week of February; spring includes three weeks in February, March, April, and one week in May; summer, the remainder of May, the whole of June and July, and one week in August; autumn occupies the rest of the year, to the first week in November, inclusive. This division, however, is not strictly accurate as to *temperature;* because the month of February is colder than November—the earth, in the latter month, being already warmed by the summer rays. The following results show the average annual differences per cent.—*plus* or *minus* the results of the tabulation on the principle of the Registrar-general. Each extends over three years, with their corresponding seasons, except the natural division, which comprehends two winters only.

| | *Smallpox.* | *Measles.* | *Scarlatina.* | *Respiratory organs.* |
|---|---|---|---|---|
| Spring .. | 28 per cent. plus. | 6 per cent. minus. | 11 per cent. minus. | 14 per cent. plus. |
| Summer. | 3 — plus. | 22 — plus. | 14 — minus. | 5 — plus. |
| Autumn. | 38 — minus. | 30 — minus. | 3 — plus. | 16 — minus. |
| Winter .. | 43 — plus. | 61 — plus. | 19 — minus. | 3 — plus. |

The table is thus read: in the metropolis, during each of the three springs of 1840-1-2, there were, on the average, 28 per cent. more deaths from smallpox than the Registrar-general's division of the seasons exhibits, 6 per cent. fewer deaths from measles, 11 per cent. fewer from scarlatina, and 14 per cent. more from diseases of the respiratory organs. In the autumns of the same three years, the deaths were not so numerous from smallpox as the Registrar-general would have us suppose, by 38 per cent.; nor from measles by 30 per cent.; nor from diseases of the respiratory organs, by 16 per cent.: but scarlatina was 3 per cent. more fatal. All these numbers, it must be remembered, are below the true difference, if *temperature* regulate the mortality, by so much as the three last weeks of November are warmer than the three last in February.

Let us pass to other points of inquiry. If the student of hygiene seeks to ascertain the influence of climate on the health and mortality, or of soil, employment, or any other of the influences before mentioned, he will derive no assistance from these Reports singly. He will never learn whether the inhabitants of the coast are more or less liable to any particular disease, or have longer or shorter lives than the inhabitants of hill-sides in the interior; nor can he compare the mortality of the latter, and their diseases, with the diseases and mortality of the people in the vales below them, or of those living on a diluvial plain, or of those on the carboniferous limestone, or on the red sandstone, &c.; because the same districts comprise an inland and a maritime, a hill-side and a valley population, &c. If he refer to the tables including the "mining" districts, to learn the most fatal diseases of colliers, he derives no specific information; for no means are given for analysis and classification of the deaths: and he knows well that the mill and the mine are generally next-door neighbours; while the employments of the factory operative and collier are too dissimilar to be ranged under the same general head.

In the Appendix to the Fifth Annual Report there is an interesting essay, by Mr. Farr, on the diseases of towns and of the open country. The mortality and diseases in five towns and seven counties, and of the metropolis and south-western division are compared. The deaths in the town districts, in the same time (four years,) out of the same amount of population, were 38 per cent. more numerous than in the country. The increase was in every class of diseases, but greatest in the zymotic class, and in that including affections of the respiratory organs.

In the present Report, Mr. Farr makes more use of algebraic formulæ than formerly. In discussing the causes of the increased mortality in towns, for example, he expresses his opinions in the form of an equation, as follows:

"$$\frac{\text{Income}}{\text{Drink} + \text{food} + \text{physic} + \text{clothing} + \text{firing} + \text{lodging} + \text{cleansing}} = r,$$

and putting $n$ for income (in money), $c$ for the aggregate cost (in money), of the seven necessaries, and $L$ for the mean physiological duration of life, the equation is $\frac{n}{c} L = L'$; $L'$ being the life in the particular circumstances. Assume the cost of a full supply of subsistence for labourers in the country to be £50 a year, that the income of the family is £50, and that he is in such favorable circumstances altogether, that he attains the natural mean term of life, the equation will be $\frac{50}{50} L = L$. But let the income be reduced to £40, and if the ratio be simple, the equation will be $\frac{40}{50} L = \cdot 8\, L$, or $\cdot 8\, L$ will be the mean duration of life of labourers in the altered circumstances." (p. 202.)

That our non-mathematical readers may understand the above (to them, probably) incomprehensible symbols, we add our explanation. By these Mr. Farr means to express his opinion, that a poor man's life is proportionate to his income. If he lives fifty years with £50 a year, deduct £10 from the cash, and ten years are deducted from the duration of his life; or, in the words of Mr. Farr, if the mean natural life = 56 years, it would be reduced to 45 years. Mr. Farr is of opinion that the ratio of mortality is as the 6th roots of the density, and gives some interesting examples. It would be an important principle if the mode of ascertaining it could be relied on, but the fallacy of such calculations is proverbial; free scope being given for assumptions, one may prove anything by figures. We are happy to see, however, that Mr. Farr very strenuously maintains that the "coefficients (the data assumed) can be determined only by careful *observation* and analysis—I mean *scientific observation*—by persons who understand the subject." Mr. Farr also suggests that the *expectation of life* should be employed to furnish exact results. In his hands, no doubt, the method would be efficient; but we apprehend there can be no valid reason for restricting the inquiry to one favorite mode. With proper corrections, the mortality amongst children might be made to exhibit the sanitary condition of a district or of a population, just as well as the expectation of life or the average age at death; or even the ratio of births might be used for hygienic calculations of this kind.

The registration of births and deaths is the registration of millions of facts in physiology and pathology, each individual fact having diversified relations. Moreover, these facts are so collected and registered (or might be), as to be capable of being tabulated, and so exhibit their diversified relations under every possible aspect. It is obvious, then, that this registration is a most important acquisition to medical science. It affords that long-wished-for desideratum, a record of multitudinous medical observations, and so similar in their essential points as to admit of comparison. In pursuance of our duty as medical journalists, we have already criticised, first, the registration of the facts, then their tabulation; we also thought it right to go a step further in pointing out one of the modes in which the defective registration might be improved, namely, by adopting Mr. Chadwick's plan: we will now suggest a method for the better tabulation of the facts.

As the registration of deaths is now conducted, the Registrar-general's efforts must necessarily be limited. One class of diseases, namely, the zymotic, may, upon the whole, be considered as registered with sufficient accuracy for scientific purposes. The deaths entered as from these diseases do not, indeed, include *all* of the class, but they include *none* of other classes. They do not include all, for perhaps one fourth of the deaths registered under other heads belong to the class. The late influenza-epidemic was not limited to the respiratory organs; it very often took the form of colitis or gastritis; sometimes it was ushered in with profuse pulmonary or intestinal hemorrhage; sometimes appeared as an apoplectic attack, andwas no doubt often the cause of the "inflammations," the "pneumonia," &c. of the registers. The deaths of children registered from "convulsions" were, in many instances, deaths from one or other of the epidemics to which children are more particularly exposed. The errors in the registration of deaths from epidemics, however, will only affect the numbers dying of these diseases. As the date of each death is given, the means of estimating the influence of meteorological changes on their development and progress are in the hands of the Registrar-general, and the estimate ought to be made, as well as on deaths generally. By meteorological changes we mean something more than changes in temperature, as we have before stated. The influence of the moon on the earth and atmosphere at her various phases, of hurricanes, electric and telluro-magnetic changes, heavy rains, floods, the seasons, ought all to be inquired into and weighed. We would not even overlook important planetary conjunctions in the inquiry; and all the necessary tabulations should be so arranged that the secondary and indirect, as well as the immediate and direct results should be ascertained; and also that the effects of one week of striking meteorological changes, or even of one day, should be compared from year to year. Thus, for example, the influence of the electric 10th of August on births and deaths should be inquired into, and the effects compared from year to year. It is quite practicable to register the hour of death; and this should be done, that the effect of diurnal meteorological changes might be ascertained.

The topographical relations of the times and causes of death can only be ascertained by local inquiries; at least, the means in the hands of the Registrar-general are so imperfect that the results would not be equivalent to the necessary labour of tabulation. In October 1842 an attempt was made by him to ascertain the sanitary condition of various registry-districts of the metropolis, and the connexion of that condition with the cleanliness, supply of water, density of population, number of persons sleeping in the same room, occupations and condition of the population, nature of their food, supply of fire, and moral habits. Questions embracing these points were issued to the district registrars; but the questions are general, and, as might be expected by any one practically acquainted with these matters, the answers are general. Indeed, they could not be otherwise, for the registrars had no means of giving the information demanded; and even if they had had the means, being generally unscientific observers, their observations must have been unscientific too. Mr. Abrahams probably displays the greatest fitness for his duties—paradoxical as it may appear by—his laconic answers, "I don't know," and "I cannot tell."

Under the existing circumstances, it appears to us that the best plan for obtaining the requisite local data, would be to secure the appointment of medical superintendent registrars, with such general scientific knowledge as may enable them to carry out a general plan of inquiry, to mark out the topographical peculiarities of their district, and conduct generally a local tabulation. The latter object might be secured by having the deaths "posted up," to use a commercial phrase, under various headings by the deputy registrars. Tables for each sub-district would be thus formed. These tables would constitute the basis of more general tables, extending over the whole district, and might be transferred with the latter to the hands of a deputy registrar-general of several counties, or to the Registrar-general himself. By this division of labour, on the true inductive method, two or three important advantages would be gained. The labour itself would be rendered comparatively trifling; the results would be ascertained and communicated to the public month by month and year by year, instead of after a delay of one or two years; a perfect system of observing the relations of meteorological and topographical influences to disease and death would be commenced, and a corps of experienced men trained up to continue it with increasing efficacy and success. The additional expense would not be more than sixty or seventy per cent. on the present outlay, and would be returned a hundredfold. A tripled expenditure, however, if compared with the advantages the nation would derive from a really efficient plan of the kind laid down, would scarcely be worthy notice. Had our space permitted a full statement of the premises, we think we could have irrefragably proved that the progress of medical science, as well as of public hygiene, is mainly dependent on a proper working of the act for the registration of births, marriages, and deaths, and may be advanced by it in a constantly accelerating ratio.

Mr. Chadwick's essay is well worthy attentive perusal. The tables have all a meaning. Some of them must have cost an immense amount of labour. One or two illustrations of the practical difficulties in the way of the commonest conveniences and comforts of town life are given. In Paris the *porteurs d'eau* effectually oppose the introduction of an increased supply of water, and therewith of the means of cleanliness and health, into every house. Mr. Whitworth, of Manchester, invented a street-sweeping machine, and tried to get it introduced into the French capital, but he found the *chiffoniers'* interest to be impregnable. At New York the great obstacle to its adoption by the "profound statesmanlike men," as Mr. Chadwick quizzically terms the political leaders there, was, that it had NO VOTES; and would displace thirty or forty "independent ones."

Mr. Chadwick wishes statists would cooperate in some general plan. We refer him to an article in our current Number (art. xiv), for a suggestion of this kind; and conclude our present one with his reflections on the subject of hygiene in general, and the facts just stated:

"The moral atmosphere under which a population is so situated is as offensive and depressing and pestilential as the physical atmosphere under which it suffers; and it is grievous to experience and melancholy to contemplate. But still there are facts of promise; we must hope and labour on; and some of the most beneficial labours of those who are fortunate in being placed above, and out of the reach of such influences, would be in the production of complete statistical returns, demonstrating, as they must do when complete, the enormous expense in money as well as in pain, sickness, and waste of life which would make it worth while to buy off on the most liberal terms every existing opposing interest to the most certain measures of human improvement.

"If there could be intercommunication and simultaneous labours of statists in different places, based on local investigation, each might afford light to advance the labours of the other, and give to vital statistics a public estimation and use beyond any which they have at present obtained!" (p. 32.)

We shall not now despair of elevating the medical profession itself to as high an estimate as Mr. Chadwick desires for vital statistics. If our readers will always remember to act on the principle that the political aggrandisement of the profession depends upon its political usefulness; and that its political usefulness must be displayed by the application of medical science to social economy—the highest and noblest use of our high and noble science—such results to civilization will follow in Great Britain as have never yet been seen in any nation.

